# New insights into the stipitate hydnoid fungi *Sarcodon*, *Hydnellum*, and the formerly informally defined *Neosarcodon*, with emphasis on the edible species marketed in Southwest China

**DOI:** 10.1186/s43008-023-00138-1

**Published:** 2024-03-26

**Authors:** Di Wang, Hui Feng, Jie Zhou, Tian-Hai Liu, Zhi-Yuan Zhang, Ying-Yin Xu, Jie Tang, Wei-Hong Peng, Xiao-Lan He

**Affiliations:** 1 Sichuan Institute of Edible Fungi, Chengdu, 610066 China; 2 Jilin Agricultural University, Changchun, 130041 China

**Keywords:** *Bankeraceae*, Cryptic species, Ectomycorrhizal, Edible mushroom, Markets, *Sarcodon imbricatus* complex, New taxa

## Abstract

*Sarcodon* and *Hydnellum* are two ectomycorrhizal genera of important ecological and economic value in Southwest China, and they are common in the free markets in this region. It was estimated that more than 1,500 tonnes of them were sold as edible per year, but there was little information about the taxonomic placements of these edible mushrooms sold in the markets. Traditional concepts of the two genera have also been challenged recently, and circumscription of *Sarcodon* and the informally defined clade “*Neosarcodon*” remained unresolved. In the present study, specimens collected in the field and purchased from the markets in Southwest China were analyzed based on morphological characters and DNA sequences. Phylogeny of the traditional *Sarcodon* s. lat. and *Hydnellum* s. lat. was reconstructed from the combined internal transcribed spacer (ITS), nuclear large ribosomal subunit (nLSU) and RNA polymerase II second largest subunit (RPB2) dataset based on expanded samples to reevaluate the taxonomic placements of the two genera. In the present molecular analyses, four distinct clades were recovered and strongly supported: *Hydnellum*, *Neosarcodon*, *Phellodon* and *Sarcodon*. *Neosarcodon* is formally introduced as a generic name to include nine species previously placed in *Sarcodon*, and the delimitation of *Sarcodon* is revised based on phylogenetic and morphological studies. Phylogenetic analyses also revealed an unexpected species diversity (17 phylogenetic species) of *Sarcodon* and *Hydnellum* in the markets; nine phylogenetic species of *Sarcodon* and eight of *Hydnellum* were uncovered from the samples collected in the markets. Eight species were resolved in the traditional *S. imbricatus* complex, with *S. imbricatus* s.str. being the most common edible stipitate hydnoid fungal species. Three of the edible *Hydnellum* species (*H. edulium*, *H. subalpinum*, and *H. subscabrosellum*), and five separated from the *S. imbricatus* complex (*Sarcodon flavidus*, *S. giganteus*, *S. neosquamosus*, *S. nigrosquamosus*, and *S. pseudoimbricatus*), are described as new. Three new Chinese records (*H. illudens*, *H. martioflavum*, and *H. versipelle*), and the notable *S. imbricatus* and *S. leucopus* are also reported.

## INTRODUCTION

*Sarcodon* and *Hydnellum* are common and important ectomycorrhizal (ECM) genera that traditionally differ in basidiome structure and are placed in *Bankeraceae*. These are stipitate hydnoid genera that both have brown tinted basidiospores, while *Sarcodon* has softer and fleshier basidiomata and *Hydnellum* ones that are hard and dry. The identification of *Sarcodon* and *Hydnellum* species was traditionally mainly based a limited range of morphological characters, such as the color of the pileus and stipe, the arrangement of the pileus surface, organoleptic features (smell and taste), and spore size (Harrison 1964, 1984; Maas Geesteranus and Nannfeldt 1969; Maas Geesteranus 1971, 1975; Baird 1985; Baird and Khan 1986; Harrison and Grund 1987; Stalpers 1993; Pegler et al. 1997; Strid 1997). However, in the last decade, DNA sequence analyses have shown the traditional generic delimitation of the two genera to be somewhat questionable (Nitare and Högberg 2012; Baird et al. 2013; Miscevic 2013; Loizides et al. 2016; Vizzini et al. 2016). Larsson et al. (2019), based on nuclear large ribosomal subunit (nLSU) sequence analysis, showed that *Hydnellum* and *Sarcodon* were distinct genera but that the current division based on basidioma texture made *Sarcodon* paraphyletic with respect to *Hydnellum*. Consequently, some species of *Sarcodon* were moved to *Hydnellum*. Additionally, a distinct clade, “*Neosarcodon*”, was informally defined in the internal transcribed spacer (ITS) sequence analysis of that study. Mu et al. (2021) also confirmed that the traditional concept of *Sarcodon* was not monophyletic, and their previously described new species of *Sarcodon* (Mu et al. 2020) were shown to be nested in *Hydnellum*. Notably, all the known species in the traditional *Sarcodon* sect. *Scabrosi* were confirmed to belong in *Hydnellum*, and the new subgenus *Hydnellum* and subgen. *Scabrosum* was established to accommodate these species (Mu et al. 2021). The traditional concept of *Sarcodon* is therefore much reduced with less than 50 species retained, but the phylogenetic relationships and monophyly of the remaining *Sarcodon* species have not been resolved using phylogenetic analyses (Larsson et al. 2019; Mu et al. 2021). Further studies based on a more extensive sampling and an expanded dataset are needed to develop more robust generic limits for these fungi.

**Fig. 1:**
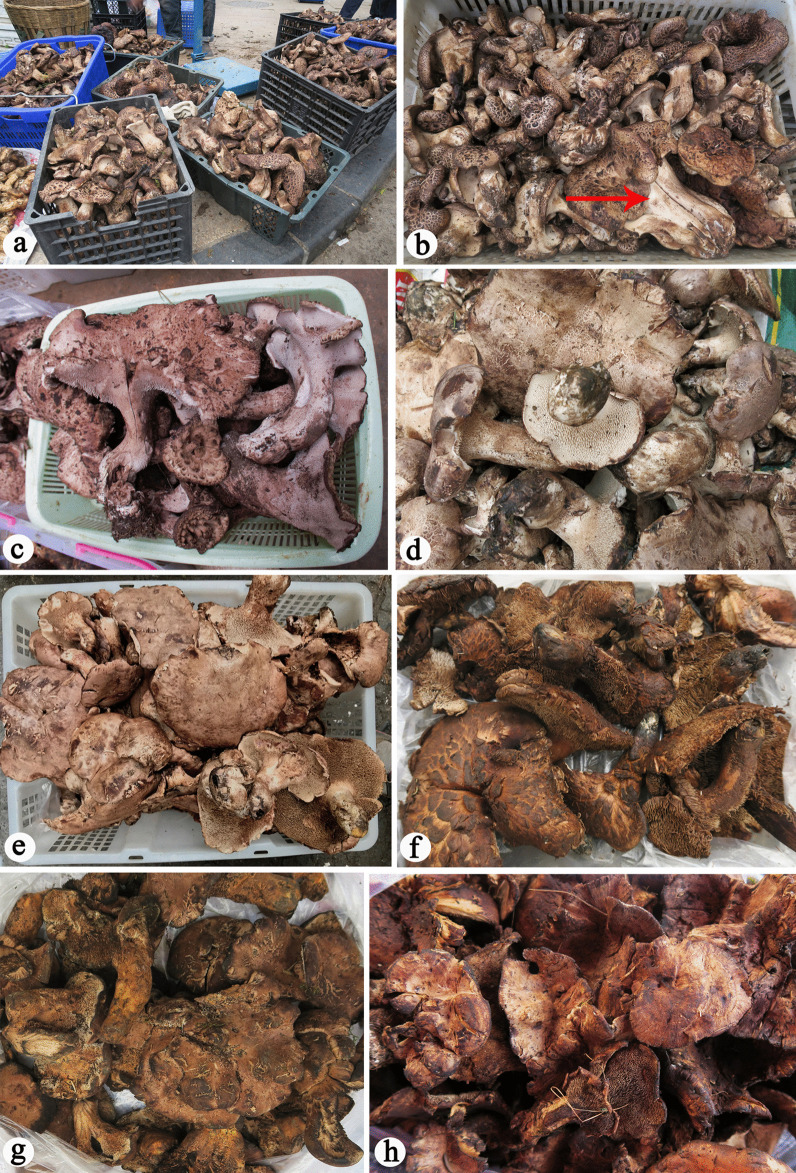
Stipitate hydnoid fungi sold in the markets in Southwest China. **a** *Sarcodon* spp. **b** *Sarcodon* spp. mixed with *Hydnellum* sp. (the red arrow indicates) **c** *Sarcodon giganteus* **d**, **e** *Sarcodon leucopus* **f** *Hydnellum illudens* **g** *H. martioflavum* **h** *H. subalpinum* mixed with *Hydnellum* spp

Although members of the stipitate hydnoid fungal genera have been considered endangered and included in the Red Data Lists of several European countries (Hrouda 1999, 2005; Walleyn and Verbeken 2000; Nitare 2006; Senn-Irlet et al. 2007), they are of important economic value in Southwest China, especially *Sarcodon* species, and are common in the free markets in this region (Fig. 1). It is estimated that more than 1,500 tonnes of *Sarcodon* species are sold in the free markets per year in Sichuan province. Studies in recent years have demonstrated the high species diversity of the stipitate hydnoid genera *Hydnellum* and *Phellodon* in China (Mu et al. 2020, 2021, 2022b; Song et al. 2022a). However, little taxonomic information about Chinese *Sarcodon* was involved in these studies. In Southwest China, *Sarcodon* species with the pileus covered by prominent squamules were usually called “hei hu zhang” or “zhang zi jun” in the markets, and were commonly identified as *S. imbricatus*, *S. aspratus* or *S. squamosus* (Dai and Li 1994; Li et al. 2015; Yang et al. 2021), among which *S. imbricatus* was the most widely used name. Although these “hei hu zhang” look the same at first glance, however, we found that these “*S. imbricatus*” specimens exhibited some subtle differences when examined closely and could represent different taxa. Molecular analyses in the laboratory confirmed this speculation.

*Hydnellum* is also common in the markets, but much less so than *Sarcodon*. Sometimes, *Hydnellum* basidiomes were found to be mixed together with “*S. imbricatus*” (Fig. 1b) and were thought to be *Sarcodon* in the traditional sense; however, these specimens actually belonged to *Hydnellum* based on comprehensive morphological and molecular analyses. In some regions of Southwest China, *Hydnellum* species were separated from *Sarcodon* s.str. and were called “jia hu zhang jun (pseudo-*Sarcodon*)” in the markets. Moreover, little information about the taxonomic placements of these *Hydnellu*m species sold in markets was documented in China. Mu et al. (2020, 2021) described 13 new *Hydnellum* species from China, most of which seemed to be different from the edible *Hydnellum* species sold in the markets. In a review of the diversity of Chinese macrofungal resources (Wu et al. 2019), only three “*Sarcodon”* species (*S. imbricatus*, *S. leucopus*, *S. violaceus*) and two *Hydnellum* species (*H. concrescens* and *H. cumulatum*) were listed as edible; *S. violaceus* has been considered as *Bankera violacea*. In other words, prior to the present study, only two edible *Sarcodon* species and two edible *Hydnellum* species were recorded in China. Additionally, it was unclear whether the names applied to these edible stipitate hydnoid fungi were correct.

In the present study, edible *Sarcodon* and *Hydnellum* specimens purchased from the markets in Southwest China, and specimens collected in the field were subjected to morphological and molecular analysis to clarify the situation. Furthermore, the phylogenetic relationships of the traditional genera *Hydnellum* and *Sarcodon* were reevaluated based on the combined ITS, nLSU and RNA polymerase II second largest subunit (RPB2) sequences, and the generic limits of *Sarcodon* s.lat. were revised based on morphological and molecular analyses.

## MATERIALS AND METHODS

### Morphological descriptions

Fresh specimens were collected in the field or markets, and our macro-morphological descriptions were based on fresh materials. The color notations followed Kornerup and Wanscher (1978). Basidiospores, basidia, and pileipellis were mounted and measured in 5% KOH, and observed using a Leica DM5000B microscope. Basidiospores were measured with ornamentation in profile view. All holotype collections are kept in the Fungarium of Sichuan Academy of Agricultural Sciences (SAAS).

### DNA extraction, polymerase chain reaction PCR amplification and sequencing

The procedures used to conduct genomic DNA extraction, PCR amplification, and sequencing were the same as in our previous study (He et al. 2013). The primers used for the ITS regions were ITS5 and ITS4 (White et al. 1990), the primers used for RPB2 amplification were rpb2-6f and rpb2-7r (Liu et al. 1999), and the nLSU regions were amplified using the primer pair LR0R and LR5 (http://www.biology.duke.edu/fungi/mycolab/primers.htm). The PCR products were sequenced in both directions.

### Sequence alignment and phylogenetic analyses

The published sequences of *Sarcodon* and *Hydnellum* downloaded from GenBank were carefully checked; those of low quality and suspect were excluded. Sequences newly generated in this study were manually corrected according to the sequence chromatograms. Phylogenetic analyses were performed using Maximum Likelihood (ML) and Bayesian analysis based on the combined ITS, nLSU, and RPB2 dataset; *Amaurodon aquicoeruleus* and *A. viridis* were used as the outgroups following Mu et al. (2021). The sequences used in this analysis are listed in Table 1 and aligned in muscle 3.6 (Edgar 2004). If necessary, the aligned sequences were manually modified employing Mega 11 (Tamura et al. 2021). All sequence data generated for this study can be accessed via GenBank: https://www.ncbi.nlm.nih.gov/genbank/. All alignments for phylogenetic analyses have been deposited in TreeBASE (http://purl.org/phylo/treebase/phylows/study/TB2:S30772). Single-locus phylogenies were constructed to detect incongruence among individual genes using the maximum likelihood (ML) method. As no conflicts were detected among the well-supported clades, the sequences of ITS, nLSU and RPB2 were combined for further analyses.

**Table 1:** Specimens sequenced or downloaded from GenBank. Sequences generated for this study are marked in bold

Species	Voucher	Source	ITS	LSU	RPB2
***Amaurodon aquicoeruleus***	**UK452**	Australia:3.7 km east of Brockman highway	**AM490944**	**AM490944**	–
***A. viridis***	**KHLarsson14947b**	Norway	**MK602707**	**MK602707**	–
***Hydnellum atrospinosum***	**Yuan 6514**	China	**MW579940**	**MW579886**	–
***H. aurantiacum***	**EBendiksen 177-07**	Norway	**MK602712**	**MK602712**	–
***H. auratile***	**J Nitare 110,926**	Sweden	MK602716	MK602716	–
***H. bomiense***	**Yuan 13,759**	**China**	**MW579941**	**MW579887**	**OK254206**
***H. brunneorubrum***	**Yuan14668**	**China**	**MW579945**	**MW579890**	**OK254218**
***H. caeruleum***	**EBendiksen 584-11**	Norway	**MK602719**	**MK602719**	–
*H. chrysinum*	SC071	–	KJ534291	–	–
*H. coactum*	**Shi181**	**China**	**MN846279**	**MN846288**	–
*H. concrescens*	REB-385	USA	JN135182	–	**KF007964**
***H. cristatum***	**REB-169**	USA: NC, GSMNP, Deep Creek	**JN135174**	–	**KF007958**
***H. cumulatum***	**REB-342**	USA: CT, Bigelow Hollow State Park	**AY569026**	–	–
***H. diabolus***	**KAH13873**	**Canada**	**AF351863**	–	–
***H. dianthifolium***	**ML61211HY**	Cyprus	**KX619419**	–	–
***H. earlianum***	**REB-375**	USA: TN, GSMNP, Gregory Ridge Tr	**JN135179**	–	**KF007962**
***H. edulium***	**SAAS 2727**	China: Sichuan Province	**OK636093**	**OP407675**	**OP434358**
***H. edulium***	**SAAS 2870**	China: Sichuan Province	**OK636094**	**OP407676**	**OP434359**
***H. edulium***	**SAAS 2920**	China: Yunnan Province	**OP437910**	**OP407677**	**OP434360**
***H. fagiscabrosum***	**GB-0195621**	**Sweden**	**MW144293**	**MW144293**	–
***H. fennicum***	**SWesterberg 110,909**	Sweden	**MK602739**	**MK602739**	–
***H. ferrugineum***	**ELarsson 356-16**	Sweden	**MK602721**	**MK602721**	–
***H. ferrugipes***	**REB-68**	USA: NC, GSMNP, Smokemont Loop Tr	**JN135176**	–	**KF007955**
***H. fibulatum***	**Yuan14646**	**China**	**MW579957**	–	–
***H. fuligineoviolaceum***	**BNylen130918**	**Sweden**	**MK602741**	**MK602741**	–
***H. fuscoindicum***	**OSC 113622**	**USA**	**EU669228**	**EU669278**	–
***H. geogenium***	**AFTOL-ID 680**	Norway	**DQ218304**	**AY631900**	**DQ408133**
***H. glaucopum***	**Edvinson110926**	**Sweden**	**MK602745**	**MK602745**	–
***H. gracilipes***	**GB-0113779**	Sweden	**MK602727**	**MK602727**	–
***H. granulosum***	**Yuan12213a**	**China**	**MW579948**	**MW579893**	**OK254213**
***H. grosselepidotum***	**Wei8120**	**China**	**MN846274**	**MN846283**	–
***H. grosselepidotum***	**SAAS2472**	**China**	**OP437911**	–	–
***H. illudens***	**SAAS 3830**	**China:** Sichuan Province	**OP437912**	**OP407678**	**OP434361**
***H. illudens***	**SAAS 3838**	**China:** Sichuan Province	**OP437913**	**OP407679**	**OP434362**
***H. illudens***	**SAAS 3844**	**China:** Sichuan Province	**OP437914**	**OP407680**	**OP434363**
***H. illudens***	**GB0195655**	**Sweden**	**MW144351**	**MW144351**	–
***H. inflatum***	**Wang80**	**China**	**MW579949**	**MW579894**	**OK254210**
***H. joeides***	**Nitare110829**	**Sweden**	**MK602751**	**MK602751**	–
***H. lepidum***	**JNitare110829**	**Sweden**	**MK602754**	**MK602754**	–
***H. lidongensis***	**Wei 8365**	China: Yunnan Province	**MN846280**	**MN846289**	–
***H. lidongensis***	**SAAS 2435**	China: Yunnan Province	**OP437915**	**OP407681**	**OP434364**
***H. lidongensis***	**Cui 18,460**	China: Tibet	**OP437916**	–	–
***H. lundellii***	**OF295814**	**Norway**	**MK602760**	**MK602760**	–
***H. martioflavum***	**OF242872**	**Norway**	**MK602761**	**MK602761**	–
***H. martioflavum***	**SAAS 2667**	**China: Sichuan** Province	**OP437917**	–	**OP434365**
***H. martioflavum***	**SAAS 2674**	**China: Sichuan** Province	**OP437918**	–	–
***H. mirabile***	**RG Carlsson 11–119**	Sweden	**MK602728**	**MK602728**	–
***H. nemorosum***	**GB-0195631**	**Sweden**	**MW144373**	**MW144373**	–
***H. parvum***	**REB-131**	**USA**	**JN135187**	–	–
***H. peckii***	**Yuan13720**	**China**	**MW579967**	**MW579906**	**OK254215**
***H. piperatum***	**REB-304**	USA: NC, GRSM, Goldmine Tr	**KC571723**	–	**KF007961**
***H. rubidofuscum***	**Yuan14654**	**China**	**MW579953**	**MW579898**	**OK254209**
***H. scabrosellum***	**GB-0195792**	**Sweden**	**MW144380**	**MW144380**	–
***H. scabrosellum***	**GB-0195807**	**Sweden**	**MW144381**	**MW144381**	–
***H. scabrosum***	**OF292320**	**Norway**	**MK602766**	**MK602766**	–
***H. scrobiculatum***	**REB-78**	**USA**	**JN135181**	–	–
***H.* sp.**	**SAAS 3824**	China: Sichuan Province	**OK636119**	–	**OP434357**
***H. spongiosipes***	**REB-107**	USA: NC, Standing Indian Campground in Nantahala NF	**KC571743**	–	**KF007957**
***H. squamulosum***	**Yuan 13,625**	**China**	**MW579956**	**MW579899**	**OK254204**
***H. suaveolens***	**E Larsson 139-09**	Norway	**MK602734**	**MK602734**	–
***H. subalpinum***	**SAAS 2778**	**China: Sichuan** Province	**OP437919**	**OP407685**	**OP434369**
***H. subalpinum***	**SAAS 2884**	**China: Sichuan** Province	**OP437920**	**OP407686**	**OP434370**
***H. subalpinum***	**SAAS 2923**	**China: Sichuan** Province	**OP437921**	**OP407687**	**OP434371**
***H. subalpinum***	**SAAS 2961**	**China: Sichuan** Province	**OP437922**	**OP407688**	**OP434372**
***H. subscabrosellum***	**SAAS 3516**	**China: Sichuan** Province	**OK636118**	**OP407682**	**OP434366**
***H. subscabrosellum***	**SAAS 3833**	**China: Sichuan** Province	**OK636110**	**OP407683**	**OP434367**
***H. subscabrosellum***	**SAAS 3842**	**China: Sichuan** Province	**OK636111**	**OP407684**	**OP434368**
***H. subsuccosum***	**REB-10**	USA: NC, Coweeta Hydrological Station, Nantahala, NF	**JN135178**	–	**KF007954**
***H. sulcatum***	**Yuan 14,521**	**China**	**MW579961**	**MW579902**	**OK254202**
***H. underwoodii***	**REB-358**	USA: TN, GSMNP, Curry Mountain Tr	**JN135189**	–	–
***H. versipelle***	**RGCarlsson13-057**	Sweden	**MK602771**	–	**MK602771**
***H. versipelle***	**SAAS 2841**	**China: Sichuan** Province	**OP437923**	**OP407689**	**OP434373**
***H. versipelle***	**SAAS 2905**	**China: Sichuan** Province	**OP437924**	**OP407690**	**OP434373**
***H. yunnanense***	**Yuan14396**	**China**	**MW579963**	**MW579904**	**OK254200**
***Neosarcodon atroviridis***	**REB 109**	USA: NC, Hidden Falls Tr in Nantahala NF	**KC571769**	–	KF007966
***N. bairdii***	**Vasco 990**	Colombia	**KR698938**	–	–
***N. colombiensis***	**Vasco 2084**	Colombia	**KP972654**	–	–
***N. pakaraimensis***	**T Henkel 9554**	Guyana: Region 7 Cuyuni-Mazaruni, Pakaraima Mountains	**KM668103**	–	–
***N. pakaraimensis***	**TH9513**	Guyana	**KC155390**	–	–
***N. pallidogriseus***	**Vasco 989**	Colombia	**KR698939**	–	–
***N. portoricensis***	**TG Baroni 8776**	Puerto Rico: Canovanas, El Yunque National Forest	**KM668100**	–	–
***N. quercophilus***	**CFMR BZ-3833**	Belize: Toledo-Cayo, Chiquibul National Park, Doyle's Delight	**NR137922**	–	–
***N. rufobrunneus***	**Vasco 1989**	Colombia	**KR698937**	–	–
***N. umbilicatus***	**CORT:011996**	Belize: Toledo-Cayo, Chiquibul National Park, Doyle's Delight	**NR137923**	–	–
***Phellodon atroardesiacus***	**Cui16951**	**China**	**MZ225632**	**MZ225597**	**MZ343197**
***P. cinereofuscus***	**Cui16962**	**China**	**MZ225583**	**MZ225605**	MZ343200
***P. griseofuscus***	**Cui18544**	**China**	**OL449265**	OL439035	OL449087
***P. melaleucus***	**Cui16242**	**China**	**OP863058**	**OP860999**	–
***P. perchocolatus***	**Cui18536**	**China**	**OL449260**	**OL439030**	–
***P. stramineus***	**Cui16959**	**China**	**MZ225588**	**NG088236**	**MZ343204**
***P. yunnanensis***	**Cui17129**	**China**	**MZ225594**	**NG088237**	**MZ343207**
***S. flavidus***	**SAAS 3832**	China: Sichuan Province	**OK636112**	**OP407699**	**OP434381**
***S. flavidus***	**SAAS 3923**	China, Sichuan Province	**OK636113**	**OP407700**	**OP434382**
***S. flavidus***	**SAAS 3805**	China: Sichuan Province	**OK636114**	**OP407697**	**OP434379**
***S. flavidus***	**SAAS 3819**	China: Sichuan Province	**OK636116**	**OP407698**	**OP434380**
***S. giganteus***	**SAAS 3576**	China: Sichuan Province	**OK636121**	**OP407701**	**OP434383**
***S. giganteus***	**SAAS 3974**	China: Sichuan Province	**OK636122**	**OP407702**	**OP434384**
***S. imbricatus***	**SSvantesson 355**	Norway	**MK602748**	**MK602748**	–
***S. imbricatus***	**SAAS 2681**	China: Sichuan Province	**OK636080**	**OP407703**	**OP434385**
***S. imbricatus***	**SAAS 2789**	China: Sichuan Province	**OK636082**	**OP407704**	**OP434386**
***S. imbricatus***	**SAAS 2859**	China: Sichuan Province	**OK636086**	**OP407705**	**OP434387**
***S. leucopus***	**O-F-296099**	Norway	**MK602755**	**MK602755**	–
***S. leucopus***	**SAAS 2875**	China, Sichuan Province	**OK636087**	**OP407707**	**OP434388**
***S. leucopus***	**SAAS 2873**	China, Sichuan Province	**OK636090**	**OP407706**	**OP434389**
***S. neosquamosus***	**SAAS 2919**	China: Yunnan Province	**OK636096**	**OP407713**	**OP434391**
***S. neosquamosus***	**SAAS 2939**	China: Yunnan Province	**OP437933**	–	–
***S. neosquamosus***	**SAAS 2914**	China: Yunnan Province	**OK636095**	**OP407712**	**OP434390**
***S. neosquamosus***	**SAAS 2926**	China: Yunnan Province	**OP437932**	–	**OP434392**
***S. nigrosquamosus***	**SAAS 3836**	China: Sichuan Province	**OK636105**	–	–
***S. nigrosquamosus***	**SAAS 3922**	China: Sichuan Province	**OK636103**	**OP407711**	–
***S. nigrosquamosus***	**SAAS 2938**	China: Sichuan Province	**OK636101**	**OP407710**	–
***S. nigrosquamosus***	**SAAS 2704**	China: Sichuan Province	**OK636106**	**OP407709**	–
***S. pseudoimbricatus***	**SAAS 2974**	China: Yunnan Province	**OK636097**	**OP407696**	–
***S. pseudoimbricatus***	**SAAS 2962**	China: Yunnan Province	**OK636098**	**OP407695**	**OP434378**
***S. pseudoimbricatus***	**SAAS 2944**	China: Yunnan Province	**OK636099**	**OP407694**	–
***S. quercinofibulatus***	**JC 20090718-2**	Italy: Girona, Puig Rodon, La Vall De Bianya	**JX271818**	**MK602773**	–
***S. scabripes***	**REB 351**	USA: NC, Marion area	**JN135191**	–	**KF007970**
***S. scabripes***	**Mushroom Observer 468,045**	USA: Arizona, Apache Co	**EU293829**	–	–
***S.* sp.3**	**SAAS 2904**	China: Sichuan Province	**OK636123**	**OP407691**	**OP434375**
***S.* sp.3**	**SAAS 3780**	China: Sichuan Province	**OK636125**	**OP407692**	**OP434376**
***S.* sp.3**	**SAAS 3976**	China: Sichuan Province	**OK636124**	**OP407693**	**OP434377**
***S.* sp.**	**SAAS 2471**	China: Sichuan Province	**OP437909**	–	–
***S. squamosus***	**O-F-177452**	Norway	**MK602768**	**MK602768**	–
***S. squamosus***	**ELarsson24812**	United Kingdom: Scotland	MK602767	MK602767	OK632671
***S. squamosus***	**OF295554**	Norway	**MK602769**	**MK602769**	–

ML analyses were carried out by the web RAxML Version 8 http://www.phylo.org/sub_sections/portal/) under the GTR + G + T model with 1000 bootstrap replicates (Miller et al. 2010; Stamatakis 2014). The “Find best tree using maximum likelihood search” option was selected when analysing. Bayesian analysis was performed using MrBayes 3.2.7 (Ronquist and Huelsenbeck 2003). The best substitution models for each marker were selected by using the Akaike Information Criterion (AIC) in jModelTest 2.1.10 (Darriba et al. 2012). The RPB2 region was treated as a single partition because that there was no introns in the studied sequences. The GTR + I + G model was selected for ITS, TIM1 + I + G for nLSU, and TrN + I + G for RPB2. Four simultaneous Markov chains were run starting from random trees, keeping one tree every 1000th generation until the average standard deviation of split frequencies was below 0.01. The burn-in value was set to discard 25% of trees when calculating the posterior probabilities. The Bayesian posterior probabilities were obtained from the 50% majority rule consensus of the trees kept. FigTree v1.4.4 (Rambaut 2018) was used to display the resulting trees.

## RESULTS

### Molecular analyses

**Fig. 2:**
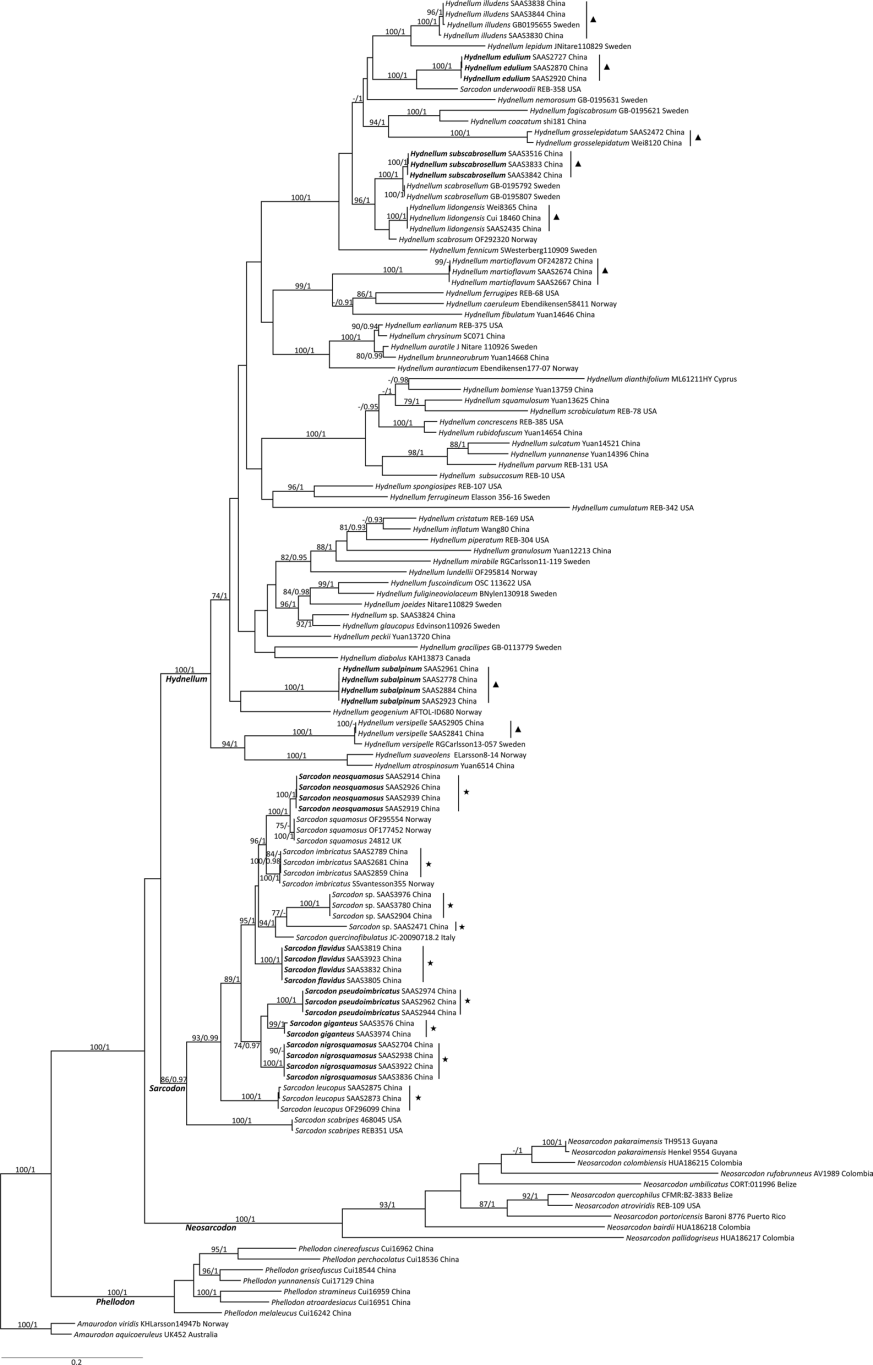
Maximum likelihood tree showing the phylogeny of *Hydnellum*, *Sarcodon* and *Neosarcodon* based on the combined nLSU, ITS and RPB2 dataset. Branches are labeled with maximum likelihood bootstrap support greater than 70% and Bayesian posterior probabilities greater than 0.95. New species are in bold. ★ indicates the *Sarcodon* species sold in Southwest China, and ▲ means the *Hydnellum* species sold in Southwest China

The analysis included 275 sequences representing 82 taxa in our analysis; 121 sequences were generated in the present study (47 ITS, 38 nLSU, and 36 RPB2 sequences). After trimming, 2763 characters were retained in the dataset, including 1140 for ITS, 946 for nLSU, and 677 for RPB2. The phylogenetic construction performed with ML and Bayesian Inference (BI) analyses for the combined dataset showed similar topology, and only the ML tree is shown in Fig. 2.

In the combined analyses, four distinct clades were recovered: *Hydnellum*, *Neosarcodon*, *Phellodon* and *Sarcodon*, all of which were strongly supported in both the ML and Bayesian analyses. Species in the *Neosarcodon* clade possessed the characters of an adnate hymenophore, an indistinct odor, and the absence of hymenial cystidia. Molecular analyses further confirmed this as a distinct group that could be treated as a genus.

The molecular analyses showed that at least 17 phylogenetic species of *Sarcodon* and *Hydnellum* were found in the markets in Southwest China: nine *Sarcodon* species (indicated by black stars ★ in the tree, Fig. 2) and eight of *Hydnellum* species (indicated by black triangles ▲ in the tree, Fig. 2). Eight of the nine edible *Sarcodon* species were grouped in the *S. imbricatus* complex, and the other as *S. leucopus*. Except for *S. imbricatus* and *S. leucopus*, the other *Sarcodon* species (*S. flavidus*, *S. giganteus*, *S. neosquamosus*, *S. nigrosquamosus*, and *S. pseudoimbricatus*) sold in the markets formed distinct clades that were different from the known species. *Sarcodon giganteus*, *S. nigrosquamosus*, and *S. pseudoimbricatus* clustered in the same clade, suggesting a close relationship. In the *Hydnellum* clade, four of the edible *Hydnellum* species were nested in the *Scabrosum* clade (*H. edulium*, *H. illudens*, *H. lidongensis*, and *H. subscabrosellum*). Among these edible *Hydnellum* species, *H. illudens*, *H. versipelle*, and *H. martioflavum* were newly recorded in China. In the tree, *H. edulium* was close to *H. underwoodii*, *H. subscabrosellum* was close to *H. scabrosellum*, while *H. subalpinum* was distant from the other *Hydnellum* species.

## Taxonomy

Based on the molecular and morphological evidence, we formally propose the clade “*Neosarcodon*” as defined by Larsson et al. (2019) as a distinct genus, *Neosarcodon*; the corresponding species should be moved from *Sarcodon* to *Hydnellum*. Accordingly, the genus description of *Sarcodon* should be revised. The distinct groups in *Hydnellum* will be redefined when more data become available.

**Fig. 3:**
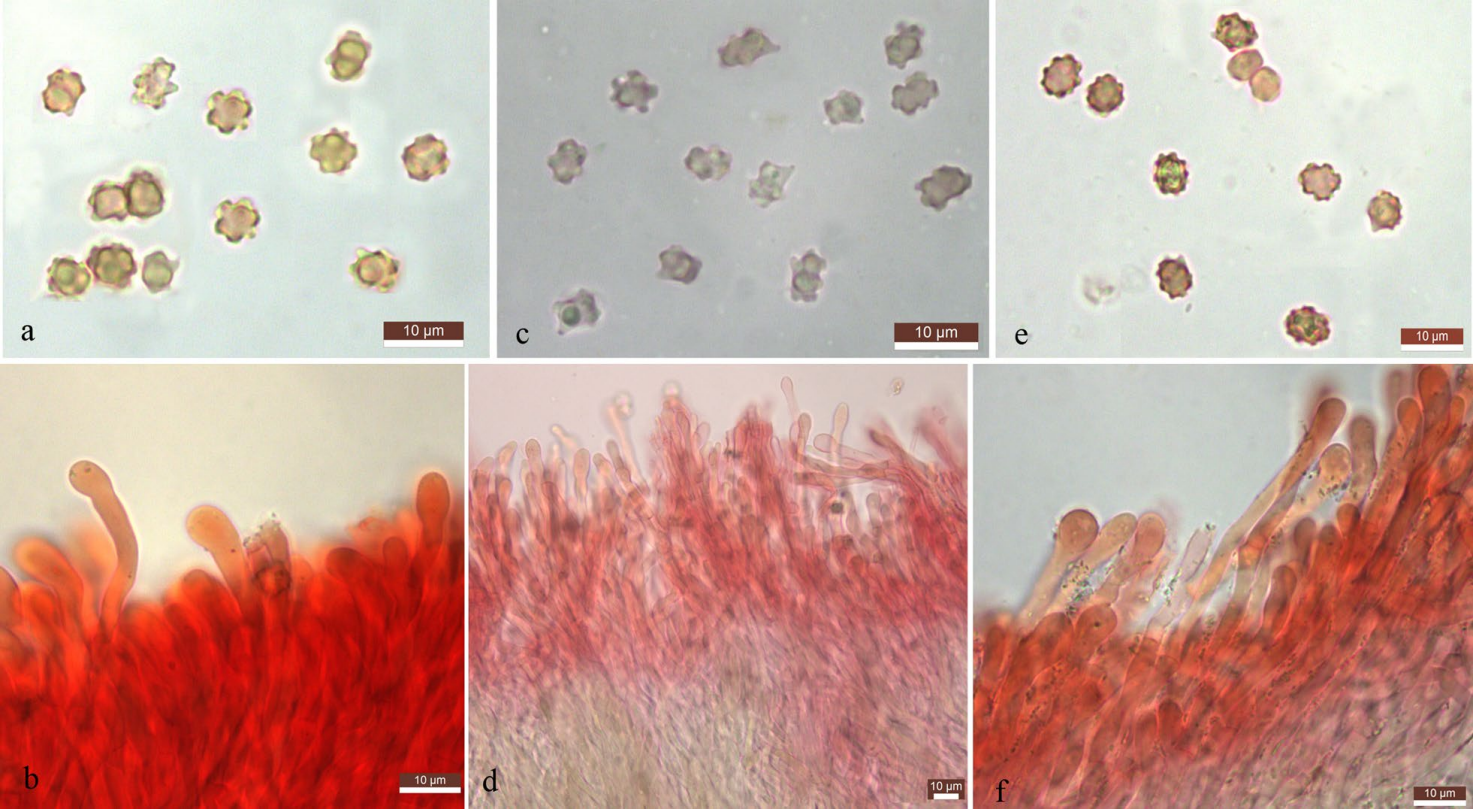
Basidiospores and cystidia of the edible *Hydnellum* species newly described in the present study. **a** Basidiospores of *H. edulium*. **b** Hymenial cystidia of *H. edulium*. **c** Basidiospores of *H. subalpinum*. **d** Hymenial cystidia of *H. subalpinum*. **e** Basidiospores of *H. subscabrosellum*. **f** Hymenial cystidia of *H. subscabrosellum*

**Fig. 4:**
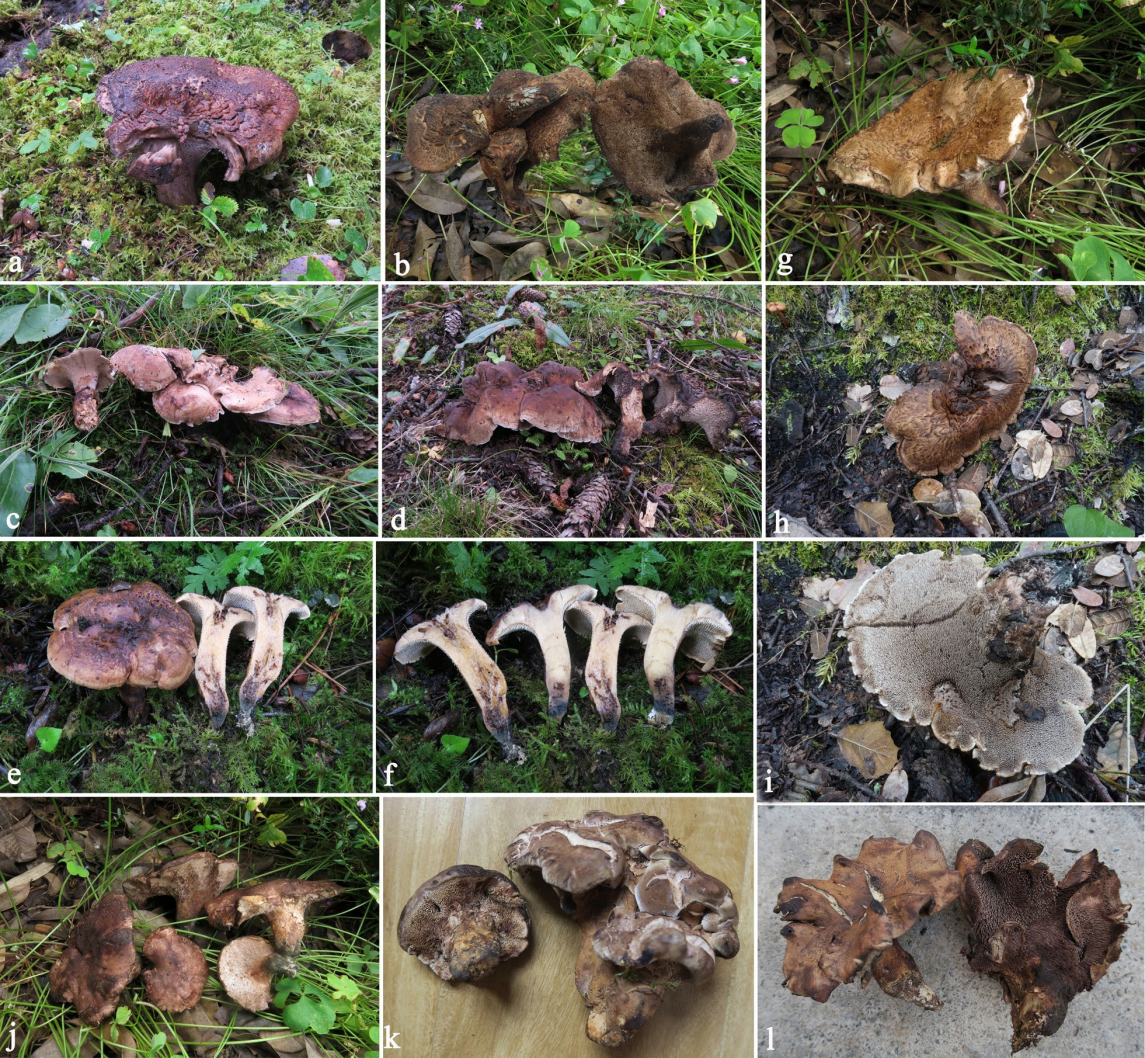
Basidiomata of edible *Hydnellum* species. **a**, **b** *H. edulium* **c**, **d** *H. subalpinum* **e**, **f** *H. subscabrosellum* **g** *H grosselepidotum* **h**, **i** *H. illudens* **j** *H. lidongensis* **k** *H. martioflavum* **l** *H. versipelle*

***Hydnellum edulium*** Xiao L. He & D. Wang, **sp. nov.**

(Figs. 3a, b, 4a, b)

MycoBank: MB 849960

*Etymology*: *edulium* (Lat.): refers to it always sold as edible mushroom in the markets.

*Diagnosis*: Differs from *Hydnellum fagiscabrosum* in the lack of a contrasting whitish pileal margin.

*Type*: **China**: *Sichuan Province*: Garze Tibetan Autonomous Prefecture, Jiulong County, 8 Sep. 2017, *He* (SAAS 2727—holotype).

*Description: Pileus* 5–9 cm diam., slightly plano-convex to planar, depressed in the center; surface brownish yellow to reddish brown; cracked by fissures forming large scales in the center, becoming depressed and small scales toward margin; scales arranged somewhat concentrically, brownish yellow to brown. *Spines* decurrent, whitish when young, pale gray to pale brownish gray when mature, becoming brown when touched, up to 0.8 cm long, spine tips whitish. *Stipe* 4–6 cm in length, 1–1.5 cm diam., paler than the pileus, becoming pale brownish when touched, with a distinctive bluish green color within the base of the stipe, slightly eccentric to central, cylindrical to attenuate below, nearly glabrous, covered by short spines in the upper stipe, solid. *Context* whitish. *Taste* mild; odor indistinct.

*Basidiospores* 4.5–5.5 × 5.5–7.0 μm, brownish, irregular in outline, ornamentation tuberculate to subechinulate, prominent, flattened to exsculpate; hilar appendage oblique. Basidia 30–42 × 6–8 μm, slender clavate, 2–4-spored, rarely 1-spored, filled with yellowish pigment, clampless. *Cystidia* numerous, 30–65 × 3.0–5.0 μm, cylindrical to slightly clavate, hyaline, clampless. *Hymenophoral* trama regular, composed of cylindrical hyphae, to 7 μm wide, septate, thin-walled, brown-yellowish, clampless. *Pileipellis* composed of cylindrical hyphae, to 17 μm wide, septate, thin-walled, with brownish epiparietal and intracellular pigment; suprapellis a cutis to subtrichoderm with some hyphae rising upward, terminal cells cylindrical to clavate, 11–20 μm diam. *Oleiferous hyphae* present. *Clamp connections* not observed.

*Ecology and distribution*: Solitary on the ground in forests dominated by *Pinus* and *Quercus*.

*Remarks*: *Hydnellum fagiscabrosum* is similar to *H. edulium*, but differs in the contrastingly whitish pileal margin. *H. edulium* and *H. illudens* are similar morphologically, however, *H. illudens* is usually found at higher elevations (> 3000 m) in subalpine areas while *H. edulium* is mainly distributed in subtropical forests at lower elevations (< 3000 m). DNA sequence analyses show that *H. scabrosum* and *H. illudens* are rather distant from *H. edulium*.

*Additional specimens examined:* **China**: *Sichuan Province*: Panzhihua City, purchased from the free market, 16 Aug. 2017, *He* (SAAS 2870); Guangyuan City, Lizhou District, Tianzhao Mountain, Sheli Tower, 14 Aug. 2017, *He* XS (SAAS 2920). *Yunnan Province*: Chuxiong, purchased from the free market, 12 Aug. 2016, *He* (SAAS 2473, 2607); Kunming City, purchased from the Mushuhua Wild Edible Mushroom Market, 21 Sep. 2017, *He* (SAAS 2700).

***Hydnellum subalpinum*** Xiao L. He & D. Wang, **sp. nov.**

**(**Figs. 3c, d, 4c, d)

MycoBank: MB849961

*Etymology*: *subalpinum* (Lat.): refers to it was discovered in the subalpine region.

*Diagnosis*: Differs from *Hydnellum cumulatum* by the white nearest margin.

*Type*: **China**: *Sichuan Province*: Garze Tibetan Autonomous Prefecture, Daofu County, Bamei country, 7 Sep. 2017, *He* (SAAS 2778—holotype).

*Description:* Basidiomata rarely single, gregarious to concrescent. Pileus 5–20 cm broad from fused pilei, planar to convex or depressed at disc, imbricate from multiple pileoli, concentric zones near margin, margin irregular to lobed from fused pilei, surface irregular and rarely smooth, generally radially rugulose to rugose, spongy tomentose to tomentose with fibrillose hairs, becoming matted or pitted; usually white nearest margin even when old, brownish yellow to brown near center. Spines decurrent, up to 8 mm long, crowded, concolorous with pileus to brown in age. Stipe 1.5–6 cm in length, 0.8–2 cm in diam., terete to subattenuate below, surface irregular from indeterminate growth, concolorous with pileus flesh. Context up to 1 cm nearest stipe, concolorous with pileus. Taste mild to none; odor not distinct.

Basidiospores 3.5–4.5 × 4–5.5 μm, irregular in outline, tuberculate, ellipsoid in profile view, brown; hilar appendage oblique. Basidia 25–38 × 5–7 μm, clavate, 4 spored; sterigmata 4–5 μm long. Cystidia numerous, 20–45 × 3.0–6.0 μm, cylindrical to clavate, hyaline. Hymenophoral trama regular, composed of cylindrical hyphae, up to 11 μm wide, septate, thin-walled, with prominent yellowish brown pigment. Pileipellis a cutis of cylindrical hyphae, up to 15 μm wide, septate, thin-walled, with brownish epiparietal and intracellular pigment, terminal cells cylindrical to clavate, 14–22 μm in diam. Oleiferous hyphae present. Clamp connections not observed.

*Ecology and distribution*: Scattered, gregarious to concrescent in forests dominated by *Abies*.

*Remarks*: Multiple fused basidiomata are diagnostic for this species. This species is most similar to *H. cumulatum* by the fused and brown basidiomata. However, *H. cumulatum* differs by bruising black, and in its occurrence in pine forest. *H. spongiosipes* is also similar, but differs in its larger basidiospores (6–7 × 5–6 μm, Baird et al. 2013).

***Additional specimens examined***: **China**: *Sichuan Province*: Garze Tibetan Autonomous Prefecture, Kangding City, Jiagenba country, 1 Sep. 2020, *He* (SAAS 4025); Daofu County, Bamei country, near Longdeng steppe, 7 Sep. 2017, *He* (SAAS 2884, 2684); purchased from the free market, 07 Sep. 2017, *He* (SAAS 2923).

***Hydnellum subscabrosellum*** Xiao L. He & D. Wang, **sp. nov**.

(Figs. 3e, f, 4e, f)

MycoBank: MB849962

*Etymology*: *subscabrosellum* (Lat.): refers to its resemblance of *H. scabrosellum*.

*Diagnosis*: Differs from *H. scabrosellum* by the much wider basidiospores.

*Type*: **China**: *Sichuan Province*: Aba Tibetan and Qiang Autonomous Prefecture, Xiaojin County, Siguniang Mt., near the gate of Shuangqiao Valley, 2 Aug. 2020, *He* (SAAS 3833—holotype).

*Description:* Pileus 3‒7 cm broad, plano-convex with a central depression, brownish yellow to reddish brown around the center, paler near margin; covered with floccose scales, almost smooth at margin. Spines strongly decurrent, up to 5 mm long, crowded, at first whitish, becoming yellowish brown when mature. Stipe 3.5‒5 cm in length, 0.8‒1.5 cm in diam., slightly eccentric to central, cylindrical to attenuate below, covered by short spines in the upper stipe, concolorous with the pileus, at the base bluish-grey or blackish-green, solid. Flesh pale yellow. Smell and taste not distinct.

Basidiospores 5.0 ‒7.0 × 4.5‒6 µm, pale brownish, irregular in outline, subglobose to ellipsoid in profile view, ornamentation tuberculate to subechinulate, prominent; hilar appendage oblique. Basidia clavate, 4-spored. Cystidia numerous, colorless, cylindrical to clavate, terminal cells 20–46 × 2.5–5.5 μm. Pileipellis composed of cylindrical hyphae, with some bundles of hyphae rising upward, thin-walled, with brownish epiparietal and intracellular pigment; terminal cells slender clavate to clavate. Oleiferous hyphae present. Clamp connections not observed.

*Ecology and distribution*: Solitary on the ground under *Pinus* and *Abies*.

*Remarks*: *H. subscabrosellum* closely resembles *H. scabrosellum* morphologically, however, the latter species has much narrower basidiospores (5.1‒6.6 × 3.4‒4.7 µm, av. = 5.8 × 4.0 µm, Nitare et al. 2021). Furthermore, steady differences between the ITS and nLSU sequences of the two species can be observed.

***Additional specimens examined*: China**: *Sichuan Province*: Aba Tibetan and Qiang Autonomous Prefecture, Xiaojin County, Siguniang Mt., near the gate of Shuangqiao Valley, 2 Aug. 2020, *He* (SAAS 3842); Xiaojin County, purchased from the free market, 1 Aug. 2020, *He* (SAAS 3835).

***Hydnellum illudens*** (Maas Geest.) Nitare, *Fungal Syst. Evol*. **7**: 245 (2021).

(Figs. 1f, 4h, i)

*Description*: Pileus 6‒10 cm, plano-convex to almost planar, somewhat depressed above the stipe, cracked by fissures forming somewhat pointed upward scales in depression, becoming small and depressed scales toward margin, ochraceous to fulvous brown, sometimes darker in the middle. Spines decurrent, up to 5 mm long, crowded, at first whitish, then becoming grayish brown when mature. Stipe 3‒5 in length, 1‒2.5 mm in diam., above concolorous with the pileus, at the base greyish blue, eccentrical to central, tapering downwards with a short rooting point, solid. Flesh pale greyish. Smell not distinct, taste slightly bitter.

Basidiospores 5.0‒6.5 × 4.5‒6.0 µm, pale brownish, subglobose to globose in outline, ornamentation tuberculate, prominent, flattened to exsculpate; hilar appendage oblique. Basidia clavate, 4-spored. Cystidia numerous, colorless, cylindrical, terminal cells 25–50 × 3–6 μm. Pileipellis composed of cylindrical hyphae, with some bundles of hyphae rising upward, thin-walled, with prominent brownish yellow epiparietal and intracellular pigment, 9–16 μm in diam. Oleiferous hyphae present. Clamp connections not observed.

*Ecology and distribution*: Solitary on ground under *Quercus*.

*Remarks*: *H. illudens*, known from Europe, is common in the free markets of Aba Tibetan and Qiang Autonomous Prefecture. Basidiospores of the Chinese collections are larger than those of European samples [4.7‒5.7 (‒6.1) × 3.5‒4.5 µm, Nitare et al. 2021]. However, the similarity of ITS sequences between them is up to 99.67%, showing that they are the same species.

*Specimens examined*: **China**: *Sichuan Province*: Aba Tibetan and Qiang Autonomous Prefecture, Xiaojin County, Meiwo country, 1 Aug. 2020, *He* (SAAS 3838); Xiaojin County, Siguniang Mt., near the gate of Shuangqiao Valley, 2 Aug. 2020, *He* (SAAS 3830); Xiaojin County, purchased from the free market, 1 Aug. 2020, *He* (SAAS 3844).

***Hydnellum martioflavum*** (Snell et al*.*) E. Larss. et al., *MycoKeys* **54**: 42 (2019).

(Fig. 1g, 4k)

*Description*: Pileus up to 9 cm broad, plano-convex to nearly planar, often depressed in the center, margin irregularly shaped, sometimes concrescent, subvelutinous to fibrillose, pale yellow brown to light brown. Spines subdecurrent, up to 5 mm long, crowded, gray whitish to gray. Stipe 3‒5 cm in length, 1‒1.8 cm in diam., central to slightly eccentric, cylindrical or attenuate below, covered by short spines in the upper stipe, subtomentose to fibrillose squamose below, concolorous with pileus, solid. Context up to 1 cm thick, white. Taste and smell not distinct.

Basidiospores 5–6.5 × 4–5.5 μm, pale brownish, irregular in outline, ornamentation tuberculate to subechinulate, prominent, flattened to exsculpate; hilar appendage oblique. Basidia 28–39 × 6–7.5 μm, clavate, unclamped, 4-spored. Cystidia numerous, colorless, cylindrical to clavate, terminal cells 20–60 × 3.0–6.0 μm. Pileipellis a cutis of cylindrical hyphae, thin-walled, up to 18 μm in diam., with yellow brownish epiparietal and intracellular pigment. Oleiferous hyphae present. Clamp connections not observed.

*Ecology and distribution*: Solitary on the ground under *Abies* and *Pinus*.

*Remarks*: ITS sequence analysis showed that these Chinese collections are *H. martioflavum*, and the basidiospores of Chinese materials are slightly larger than those of specimens collected from the United States [(4) 5–6 × 3–5 μm, Baird et al. 2013].

*Specimens examined*: **China**: Sichuan Province: Aba Tibetan and Qiang Autonomous Prefecture, Maerkanga County, purchased from the free market, 26 Sep. 2017, *He* (SAAS 2667, 2674).

***Hydnellum versipelle*** (Fr.) E. Larss. et al*.*, *MycoKeys* **54**: 42 (2019).

(Fig. 4l).

*Description*: Pileus up to 20 cm broad, convex to planar or depressed, sometimes irregular, subsquamulose to fibrillose or pubescent, margin often lobed or irregular, brownish orange to reddish brown. Spines decurrent, up to 0.9 cm long, crowded, whitish when young, becoming darker when mature, white on tips. Stipe 4–8 cm in length, 0.8–1.2 cm in diam., central to slightly eccentric, cylindrical, terete to attenuate below, subsquamulose, fibrillose, concolorous with pileus, solid. Context up to 1 cm thick nearest stipe, white. Taste and odor not distinct.

Basidiospores 4–5.5 × 3–4 μm, pale brownish, irregular in outline, ornamentation tuberculate, prominent, flattened to exsculpate; hilar appendage oblique. Basidia 25–38 × 5–7 μm, clavate, clamped, 4-spored. Cystidia numerous, colorless, cylindrical to slightly clavate, terminal cells 26–45 × 3–5.5 μm. Pileipellis composed of cylindrical hyphae, up to 17 μm in diam., clamped, with brownish epiparietal and intracellular pigment. Oleiferous hyphae present. Clamp connections present.

*Ecology and distribution*: Solitary on the ground under *Abies*.

*Remarks*: *H. versipelle*, known from northern portions of the United States and Europe, was found to be sold mixed with *H. subalpinum* in Sichuan province, where they were called “Jia Hu Zhang Jun”. The morphological characteristics of Chinese materials matched those of materials collected from the southeastern United States; and only one different base was observed between the ITS sequences of Chinese and European collections (MK602770, MK602771 and MK602772).

*Specimens examined*: **China**: *Sichuan Province*: Garze Tibetan Autonomous Prefecture, Daofu County, Bamei country, in the wayside near Longdeng steppe, 16 Sep. 2017, *He* (SAAS 2841, 2905).

***Sarcodon*** Quél. ex P. Karst., *Revue Mycol*. **3**(9): 20 (1881).

*Description*: Terrestrial with stipitate pileus. Pileus with multiple shapes, breaking into appressed or areolate scales, sometimes nearly smooth. Stipe solid, sometimes hollow at the stipe base, concolorous with pileus or slightly paler. Spines usually decurrent, dirty white or pallid at first, later with some shade of brownish gray. Context fleshy, soft, brittle, whitish to pale grayish. Odor strong but agreeable especially upon drying, taste bitter or indistinct. Hyphae inflated and thin-walled, clamp connections numerous. Basidiospores generally subglobose, tuberculate, brown in mass. Hymenial cystidia numerous, cylindrical to narrowly clavate.

***Sarcodon flavidus*** Xiao L. He & D. Wang, **sp. nov**.

(Figs. 5a-c, 6a, b)

**Fig. 5:**
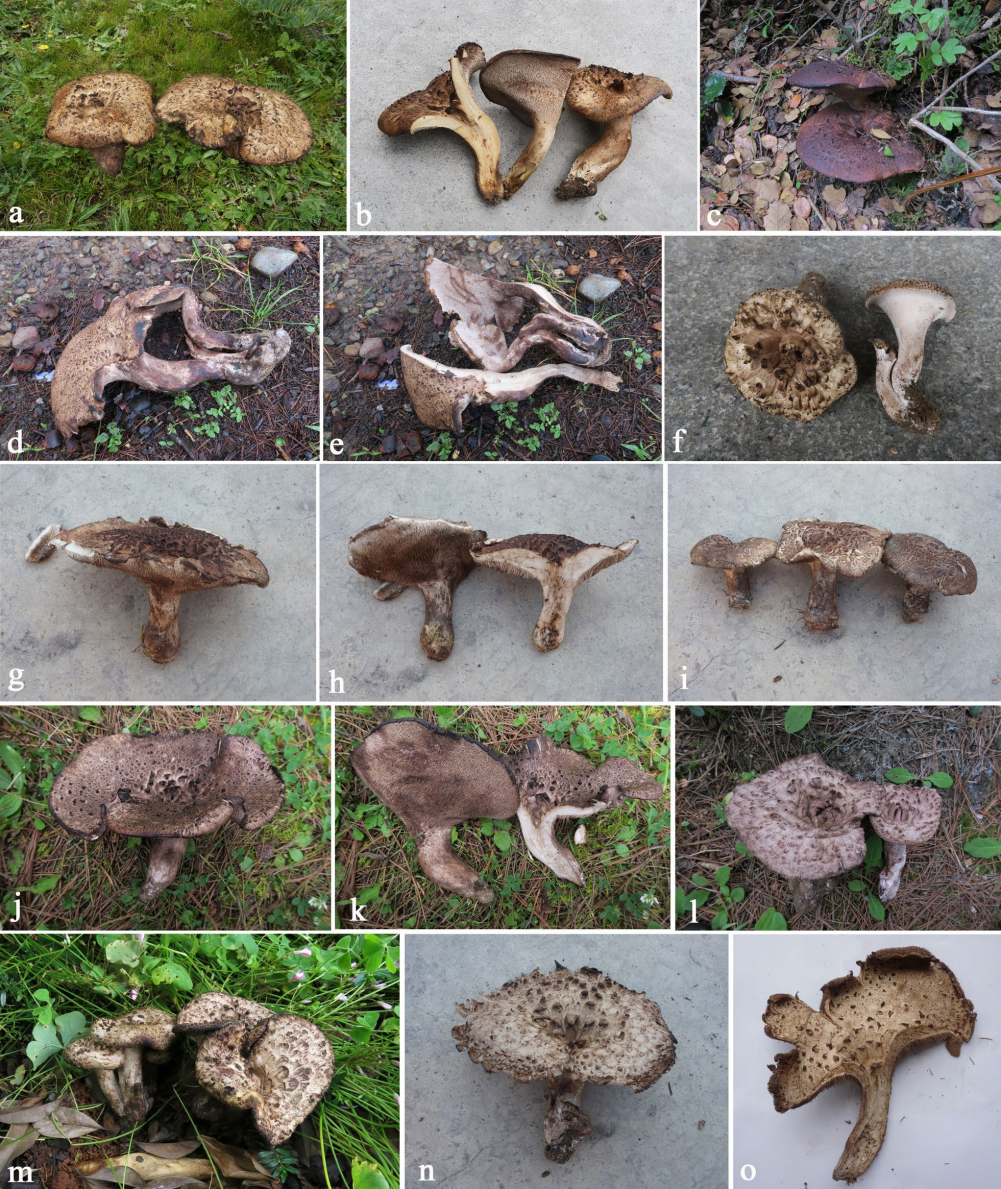
Basidiomata of edible *Sarcodon* species newly described in the present study. **a-c** *Sarcodon flavidus* **d-f** *S. giganteus* **g-i** *S. neosquamosus* **j-l** *S. nigrosquamosus* **m–o** *S. pseudoimbricatus*

**Fig. 6:**
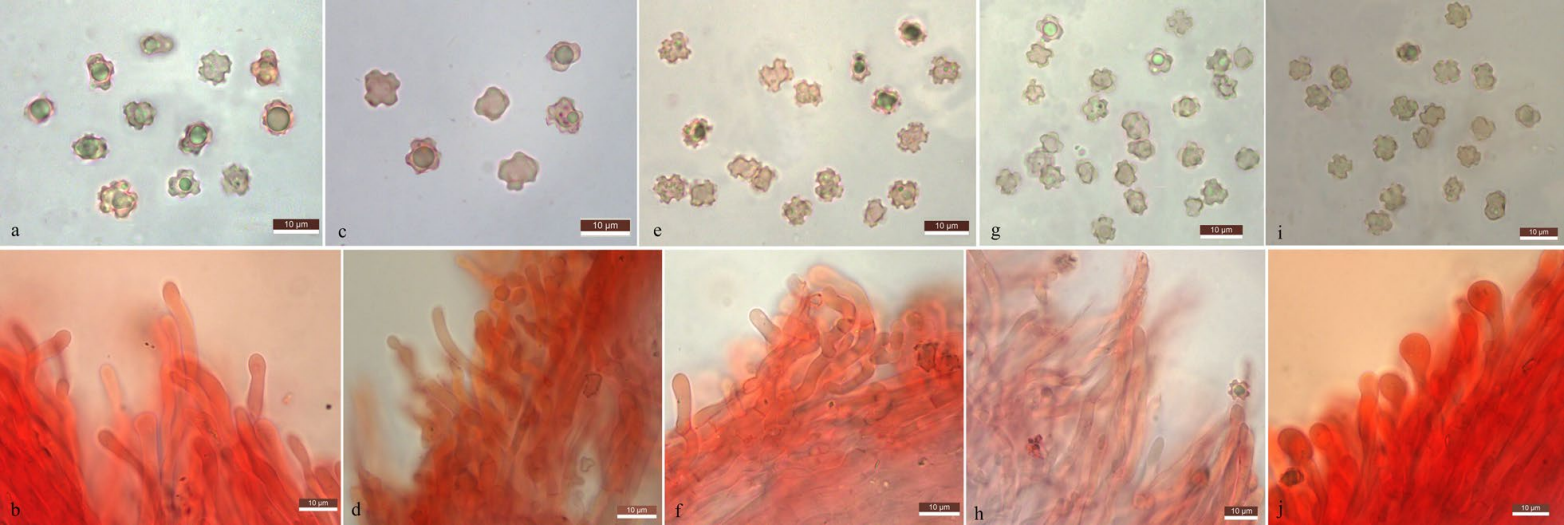
Basidiospores and cystidia of the edible *Sarcodon* species newly described in the present study. **a** Basidiospores of *S. flavidus*. **b** Hymenial cystidia of *S. flavidus*. **c** Basidiospores of *S. giganteus*. **d** Hymenial cystidia of *S. giganteus*. **e** Basidiospores of *S. neosquamosus*. **f** Hymenial cystidia of *S. neosquamosus*. **g** Basidiospores of *S. nigrosquamosus*. **h** Hymenial cystidia of *S. nigrosquamosus*. **i** Basidiospores of *S. pseusoimbricatus*. **j** Hymenial cystidia of *S. pseusoimbricatus*

MycoBank: MB849963

*Etymology*: *flavidus* (Lat.): refers to the yellow pileus.

*Diagnosis*: Differs from *Sarcodon imbricatus* by the yellowish pileus.

*Type*: **China**: *Sichuan Province*: Garze Tibetan Autonomous Prefecture, Kangding City, Jiagenba country, 1 Sep. 2020, *He* (SAAS 3725—holotype).

*Description*: Pileus 7–12 cm in diam., plano-convex, depressed in the center; surface yellow, becoming brownish yellow when touched; cracked by fissures forming large and coarse scales in the center, becoming depressed small and floccose scales toward margin; scales arranged concentrically, brownish yellow to brown. Spines decurrent, whitish when young, pale gray to pale brownish gray when mature, becoming brown when touched, up to 0.8 cm long. Stipe 2.5–4 cm in length, 1–1.5 cm in diam., concolorous with the pileus, becoming pale brownish when touched, slightly eccentric to central, cylindrical to attenuate below, nearly glabrous, covered by short spines in the upper stipe, solid. Context pale yellowish. Taste mild; smell strong but agreeable when dried.

Basidiospores 6.5–8.5 × (4.5) 5.0–7.0 μm, brownish, irregular in outline, ornamentation tuberculate, prominent, flattened to exsculpate; hilar appendage oblique. Basidia 30–39 (–45) × 6.5–8 μm, slender clavate, 4-spored, hyaline, clamped. Cystidia numerous, 25–60 × 3.0–6.0 μm, cylindrical, hyaline, clamped. Hymenophoral trama regular, composed of cylindrical hyphae, up to 8 μm wide, septate, thin-walled, yellowish brown, clamped. Pileipellis composed of cylindrical hyphae, up to 15 μm wide, septate, thin-walled, with brownish epiparietal and intracellular pigment; suprapellis a cutis to subtrichoderm with some hyphae rising upward, terminal cells cylindrical to clavate, 14–22 μm in diam. Oleiferous hyphae present. Clamp connections abundant.

*Ecology and distribution*: Solitary, scattered or in small clusters on the ground in mixed forests of *Abies*, *Pinus* and *Quercus*.

*Remarks*: This species is distinguished by the yellowish pileus, which became brownish yellow when touched. *Sarcodon flavidus* is morphologically similar to *S. imbricatus*, but the latter differs in the darker pileus covered by imbricate and larger scales, as well as larger spores. *S. scabrous* and *S. underwoodii* are somewhat similar to *S. flavidus* morphologically; however, the two species have been proved to be members of *Hydnellum*.

*Additional specimens examined*: **China**: *Sichuan Province*: Garze Tibetan Autonomous Prefecture, Kangding City, purchased from the free market, 07 Aug. 2020, *He* (SAAS 3914, 3923, 3805, 3819).

***Sarcodon giganteus*** Xiao L. He & D. Wang, **sp. nov**.

(Figs. 5d-f, 6c, d)

MycoBank: MB 849964

*Etymology*: *giganteus* (Lat.): refers to the large basidiomata.

*Diagnosis*: Separated from *S. imbricatus* by the larger basidiomata, the grayish white to beige pileus covered with small scales.

*Type*: **China**: *Sichuan Province*: Liangshan Yi Autonomous Prefecture, Xichang County, Daqing country, 14 Jul. 2020, *He* (SAAS 3576—holotype).

*Description*: Pileus 12–40 cm in diam., fan-shaped, usually depressed above the stipe; surface grayish white to beige; cracked by fissures forming somewhat upward-pointed scales in depression, becoming small scales toward margin; scales brownish yellow, becoming brown when touched. Spines decurrent, grayish white when young, pale gray to brownish gray when mature, becoming brown when touched, up to 1.2 cm long. Stipe 7–10 cm in length, 1.5–2 cm in diam., concolorous with the pileus, becoming brown when touched, eccentric to lateral, cylindrical to attenuate below, nearly glabrous, covered by short spines in the upper stipe, solid. Context grayish white; taste mild; smell strong but agreeable when dried.

Basidiospores 6.5–7.5 × (4.5) 5–6.5 μm, brownish yellow, irregular in outline, ornamentation tuberculate, prominent, flattened to exsculpate; hilar appendage oblique. Basidia 25–35 × 6.5–8 μm, slender clavate, 2–4-spored, hyaline, clamped. Cystidia numerous, cylindrical to slightly clavate, 15–35 × 3–5 μm, hyaline, clamped. Hymenophoral trama regular, composed of cylindrical hyphae, 3–8 μm in diam., pale yellowish, clamped. Pileipellis composed of cylindrical hyphae, with some bundles of hyphae rising upward, thin-walled, clamped, with brownish yellow epiparietal and intracellular pigment; terminal cells slender clavate to clavate, 14–22 μm in diam. Oleiferous hyphae present. Clamp connections abundant in all tissues.

*Ecology and distribution*: Scattered or in small clusters on the ground in forests dominated by *Pinus* and *Quercus*.

*Remarks*: *S*. *giganteus* is distinguished from the other *Sarcodon* species by its rather large and paler basidiomata when mature. The pale and sparsely distributed scales are different from the most common *S. imbricatus*. However, the young* S*. *giganteus* is hardly to separate from the other *Sarcodon* species based on the morphological characters. ITS and RPB2 sequences of *S*. *giganteus* and the similar species are quite different.

*Additional specimens examined*: **China**: *Sichuan Province*: Liangshan Yi Autonomous Prefecture, Xichang City, purchased from the free market, 08 Aug. 2020, *He* (SAAS 3974); Dechang County, purchased from the free market, 18 Aug. 2021, *He* (SAAS 4026).

***Sarcodon neosquamosus*** Xiao L. He & D. Wang, **sp. nov**.

(Figs. 5g-i, 6e, f)

MycoBank: MB 849985

*Etymology*: *neosquamosus* (Lat.): refers to resemblance of *S. squamosus*.

*Diagnosis*: Differs from *Sarcodon squamosus* by the reddish brown pileus.

*Type*: **China**: *Yunnan Province*: Lanping County, Tongdian country, 19 Sep. 2017, *He* (SAAS 2926—holotype).

*Description*: Pileus 6–11 cm in diam., plano-convex to nearly planar, not depressed or slightly depressed in the center; surface reddish brown; covered by coarse scales with suberect tips, becoming small depressed floccose scales toward the margin; scales concolorous with the background at first, becoming dark brown with age. Spines usually short decurrent, grayish white when young, pale gray when mature, becoming brown when touched, up to 0.6 cm long. Stipe 3–5 cm in length, 0.8–1.4 cm in diam., pale grayish, becoming brownish when touched, usually eccentric, cylindrical and somewhat inflated at the base, solid. Context whitish to grayish white; taste mild; smell strong but agreeable when dried.

Basidiospores 6.5–8.5 × 5–7.0 μm, brownish yellow, irregular in outline, ornamentation tuberculate, prominent, flattened to exsculpate; hilar appendage oblique. Basidia 25–35 × 6.5–8 μm, slender clavate, 2–4-spored, hyaline, clamped. Cystidia numerous, cylindrical to clavate, 25–50 × 3.5–6.5 μm, hyaline, clamped. Hymenophoral trama regular, composed of cylindrical hyphae, 3–8 μm in diam., pale yellowish, clamped. Pileipellis composed of cylindrical hyphae, with some bundles of hyphae rising upward, thin-walled, clamped, with brownish yellow epiparietal and intracellular pigment; terminal cells slender clavate to clavate, 14–22 μm in diam. Oleiferous hyphae present. Clamp connections abundant in all tissues.

*Ecology and distribution*: Solitary on the ground under *Pinus*.

*Remarks*: *S. squamosus* is the most similar species to *S. neosquamosus*, and it is difficult to separate the two species based on the pileal morphological characters alone. According to Johannesson et al. (1999), *S. squamosus* has a yellowish brown to vinaceous brown pileus, the spines are often with a tint of greyish blue when fresh, the stipe is attenuated at the base, and the context is whitish but sometimes blackish brown in the stipe base. Additionally, the partial LSU sequences and complete ITS sequences of *S. squamosus*, published by Johannesson et al. (1999), Vizzini et al. (2013) and Larsson et al. (2019), are different from those of *S. neosquamosus*. *S. imbricatus* is separated from *S. neosquamosus* by its brown pileus with a more prominent center depression, as well as its occurrence in spruce forest.

*Additional specimens examined*: **China**: *Yunnan Province*: Lanping County, Tongdian country, 19 Sep. 2017, *He* (SAAS 2939); Kunming City, purchased from the free market, 21 Sep. 2017, *He* (SAAS 2914, 2919).

***Sarcodon nigrosquamosus*** Xiao L. He & D. Wang, **sp. nov**.

(Figs. 5j-l, 6g, h)

MycoBank: MB 849983

*Etymology*: *nigrosquamosus* (Lat.): refers to the almost black scales on the pileus when mature.

*Diagnosis*: Differs from *Sarcodon imbricatus* by having relatively larger pileus, dark smaller and denser pileal scales, and relatively slender stipe.

*Type*: **China**: *Sichuan Province*: Garze Tibetan Autonomous Prefecture, Luding County, Erlang Mountain, 07 Aug. 2020, *He* (SAAS 3827—holotype).

*Description*: Pileus 8–20 cm in diam., plano-convex to nearly planar, depressed in the center; surface whitish gray to pale brown; cracked by fissures forming large and coarse scales with tips pointed upward in the center, becoming small depressed floccose scales toward the margin; scales concolorous with the background at first, becoming darker with age, tips often brown to black when mature. Spines decurrent, grayish white when young, pale gray to brownish gray when mature, becoming brown when touched, up to 0.7 cm long. Stipe 3–5.5 cm in length, 1–1.6 cm in diam., pale brownish yellow to concolorous with the pileus, eccentric to central, occasionally lateral, cylindrical to attenuate below, covered by short spines in the upper stipe, solid. Context grayish white; taste mild; smell strong but agreeable when dried.

Basidiospores 6.0–8.0 × 5.0–6.0 μm, brownish yellow, irregular in outline, ellipsoid in profile view, ornamentation tuberculate, prominent, flattened to exsculpate; hilar appendage oblique. Basidia 25–35 × 6.5–8 μm, slender clavate, 2–4-spored, hyaline, clamped. Cystidia numerous, cylindrical to slightly clavate, 25–50 × 3.5–6.5 μm, hyaline, clamped. Hymenophoral trama regular, composed of cylindrical hyphae, 3–8 μm in diam., pale yellowish, clamped. Pileipellis composed of cylindrical hyphae, with some bundles of hyphae rising upward, thin-walled, clamped, with brownish yellow epiparietal and intracellular pigment; terminal cells slender clavate to clavate, 14–22 μm in diam. Oleiferous hyphae present. Clamp connections abundant in all tissues.

*Ecology and distribution*: Solitary on the ground under *Pinus* and *Quercus*.

*Remarks*: *Sarcodon nigrosquamosus* is separated from *S. imbricatus* by having relatively larger pileus, smaller and denser pileal scales and a relatively slender stipe. *S. aspratus* and *S. squamosus* were usually treated as synonyms of *S. imbricatus*, however, several studies based on ITS sequences have shown that they are different species (Johannesson et al. 1999; Vizzini et al. 2013). According to Johannesson et al. (1999), *S. squamosus* has a yellowish brown to vinaceous brown pileus. The published ITS sequences labeled as *S. aspratus* and *S. squamosus* in GenBank are different from *S. nigrosquamosus*.

*Additional specimens examined*: **China**: *Sichuan Province*: Garze Tibetan Autonomous Prefecture, Kangding City, purchased from the free market, 08 Aug. 2020, *He* (SAAS 3836, 3922); Jiulong County, purchased from the free market, 08 Sep. 2017, *He* (SAAS 2758). *Yunnan Province*: Nanhua County, purchased from the free market, 19 Sep. 2017, *He* (SAAS 2960, 2938, 2704).

***Sarcodon pseudoimbricatus*** Xiao L. He & D. Wang, **sp. nov**.

(Figs. 5m-o, 6i, j)

MycoBank: MB 849984

*Etymology*: *pseudoimbricatus* (Lat.): refers to resemblance of *S. imbricatus*.

*Diagnosis*: Differs from *Sarcodon imbricatus* by the paler colored pileus and scales when mature.

*Type***: China**: *Yunnan Province*: Nanhua County, purchased from the free market, 19 Sep. 2017, *He* (SAAS 2962—holotype).

*Description*: Pileus 7–15 cm in diam., plano-convex to nearly planar, depressed in the center; surface whitish; cracked by fissures, forming large and sparse scales with tips pointed upward in the center, becoming small scales toward the margin;scales concolorous with the background at first, tips often darker when mature. Spines strongly decurrent, grayish white to pale gray, becoming brownish when touched, up to 0.7 cm long. Stipe 3–5 cm in length, 1–1.3 cm in diam., concolorous with the pileus, eccentric, occasionally lateral, cylindrical to attenuate below, covered by short spines in the upper stipe or almost the entire stipe, solid. Context grayish white; taste mild; smell strong but agreeable when dried.

Basidiospores 6–7.5 × 4.5–5.5 (6) μm, brownish yellow, irregular in outline, ornamentation tuberculate, prominent, flattened to exsculpate; hilar appendage oblique. Basidia 27–41 × 6–7.5 μm, slender clavate, 4-spored, hyaline, clamped. Cystidia numerous, cylindrical to clavate, 27–50 × 3–7.5 μm, hyaline, clamped. Hymenophoral trama regular, composed of cylindrical hyphae, 3–8 μm in diam., yellowish, clamped. Pileipellis composed of cylindrical hyphae, 5–12 μm in diam., thin-walled, clamped, with yellowish epiparietal and intracellular pigment. Oleiferous hyphae present. Clamp connections present in all tissues.

*Ecology and distribution*: Growing in small clusters or solitary on the ground in forests dominated by *Pinus*.

*Remarks*: *S. imbricatus* is the most similar species to *S. pseudoimbricatus*. However, *S. pseudoimbricatus* is separated from *S. imbricatus* by having a relatively paler pileus and scales.

*Additional specimens examined*: **China**: *Yunnan Province*: Chuxiong City, purchased from the free market, 20 Sep. 2017, *He* (SAAS 2974); Nanhua County, purchased from the free market, 19 Sep. 2017, *He* (SAAS 2944).

***Sarcodon imbricatus*** (L.) P. Karst., *Revue mycol*., Toulouse **3**(9): 20 (1881).

(Figs. 7a-c)

**Fig. 7:**
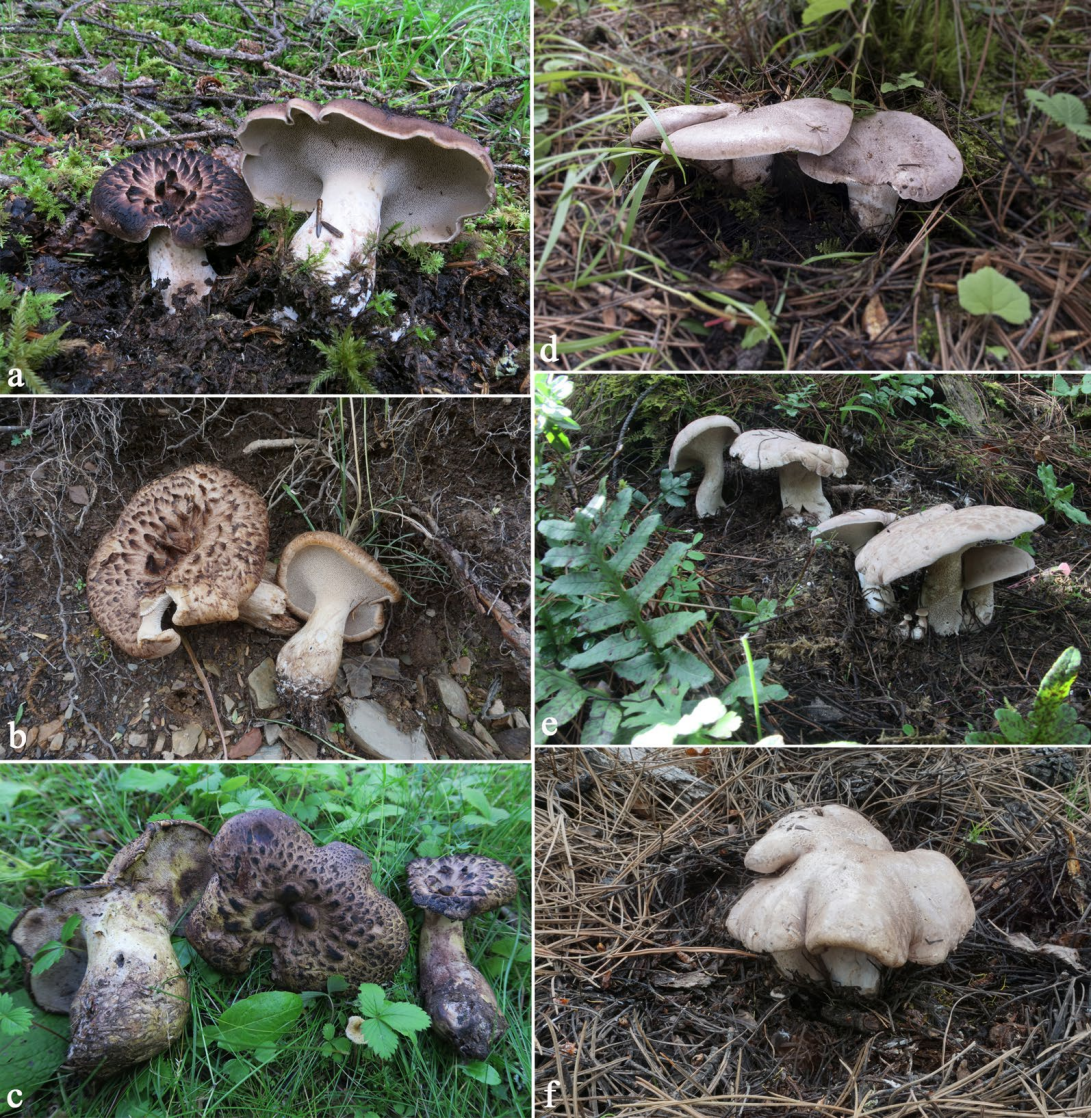
Basidiomata of the two most common *Sarcodon* species in markets. **a-c** *Sarcodon imbricatus*** d-f** *S. leucopus*

*Description*: Pileus 6–22 cm in diam., plano-convex, sometimes irregular, depressed in the center; surface brownish yellow; covered by coarse scales with tips suberect; scales concolorous with the background at first, becoming dark brown with age. Spines decurrent, grayish white when young, pale gray when mature, becoming brown when touched, up to 0.9 cm long. Stipe 2.5–6 cm in length, 0.8–1.6 cm in diam., pale grayish, becoming brownish or greenish when touched sometimes, usually eccentric, cylindrical to attenuate below, solid. Context grayish white; taste mild; smell strong but agreeable when dried.

Basidiospores 6.5–8.5 × 5.5–6.5 µm, brownish yellow, irregular in outline, ornamentation tuberculate, prominent, flattened to exsculpate; hilar appendage oblique. Basidia 25–35 × 6.5–9 μm, slender clavate, 4-spored, hyaline. Cystidia numerous, cylindrical to clavate, hyaline. Hymenophoral trama regular, composed of cylindrical hyphae, pale yellowish. Pileipellis composed of cylindrical hyphae, with some terminal cells upward, thin-walled. Oleiferous hyphae present. Clamp connections abundant in all tissues.

*Ecology and distribution*: Solitary or scattered on the ground under fir-spruce forests.

*Remarks*: According to the identification of 132 specimens, *S. imbricatus* is the most common hydnoid species in the free markets. Examination of macro-morphological characters showed that some collections of *S. imbricatus* staining greenish when touched (Fig. 7c). This character was not mentioned in the previous literature, and we thought these collections to be a different species from *S. imbricatus*, however, molecular evidence proved that they are identical to *S. imbricatus*.

*Specimens examined*: **China**: *Sichuan Province*: Kangding City, Jiagenba country; 1 Sep. 2020, *He* (3802); Daofu County, purchased from the free market, 21 Jul. 2017, *He* (SAAS 859); Yajiang County, Milongcountry, 5 Aug. 2019, *Wang* (SAAS 3121); Maerkang City, purchased from the free market, 26 Jul. 2017, *He* (SAAS 2681, 2789).

***Sarcodon leucopus*** (Pers.) Maas Geest. & Nannf., *Svensk Bot. Tidskr*. **63**: 415 (1969).

(Figs. 1d, e, 7d-f)

*Description*: Pileus 6–20 cm in diam., plano-convex to nearly planar, sometimes irregular; surface grayish white, becoming somewhat grayish purple when touched; almost smooth, somewhat velutinous and chapped. Spines decurrent, grayish white when young, pale gray when mature, becoming brown when touched, up to 0.9 cm long. Stipe 3–7 cm in length, 0.8–2 cm in diam., pale grayish, becoming grayish purple when touched, usually eccentric, cylindrical to attenuate below, solid. Context grayish white; taste rather bitter; smell strong but agreeable when dried.

Basidiospores 6–7.5 × 5–6 µm, brownish yellow, irregular in outline, ornamentation tuberculate, prominent, flattened to exsculpate; hilar appendage oblique. Basidia 25–37 × 6–8 μm, slender clavate, 4-spored, hyaline. Cystidia numerous, cylindrical to clavate, 27–53 × 3–7 μm, hyaline. Hymenophoral trama regular, composed of cylindrical hyphae, 3–8 μm in diam., pale yellowish, clamped. Pileipellis composed of cylindrical hyphae, thin-walled. Oleiferous hyphae present. Clamp connections abundant in all tissues.

*Ecology and distribution*: Solitary or gregarious on the ground under pine forests, also found in forests of *Picea* and *Quercus*.

*Remarks: S. leucopus* could be discovered in both pine forests and spruce-oak forests. ITS sequences of the specimens collected in China were almost identical to those of Larsson et al. (2019). In Mu et al. (2020), *S. leucopus* was documented as a new Chinese record based on molecular evidence, however, no pictures or descriptions based on the Chinese materials was provided. Mleczko et al. (2011) has ever described this species in detail, and some descriptions in that study were different from the morphological characters observed in specimens collected from China. In Mleczko et al. (2011), pileus of *S. leucopus* was covered with brownish olive scales, and basidiospore measurement was (7) 8–10 × 7–9 µm. The Chinese materials possess almost smooth pileus; basidiospores are much smaller. The most distinct character of *S. leucopus* is the rather bitter taste, just like bitter gourd. It was sold as edible in Sichuan Province, and common in Yajiang, Jiulong and Muli Counties.

*Specimens examined*: **China**: *Sichuan Province*: Muli County, Yeerhong country, 21 Jul. 2016, *He* (SAAS 2201); Jiulong County, purchased from the free market, 8 Sep. 2017, *He* (SAAS 2731); Daocheng County, Yeerhong country, 4 Aug. 2022, *He* (SAAS 4152).

***Neosarcodon*** Xiao L. He, Di Wang & W.H. Peng, **gen. nov.**

MycoBank: MB 849989

*Etymology*: “*Neo*-” refers to the resemblance of *Sarcodon*.

*Type: Neosarcodon pakaraimensis* (A. Grupe & T.W. Henkel) Xiao-L. He et al.

Basidiomata terrestrial with stipitate pileus. Pileus conic, broadly conic to plano-convex or nearly planar with age, never infundibuliform, surface smooth to fibrillose. Stipe hollow to solid, concolorous with pileus or slightly paler. Spines adnate, white or pallid at first, later with some shade of brownish gray. Context fleshy, soft, brittle, whitish to pale grayish. Odor indistinct. Hyphae inflated and thin-walled, clamp connections numerous. Basidiospores generally subglobose, tuberculate, brown in mass. Hymenial cystidia absent.

These previously published species are transferred to *Neosarcodon*:

***Neosarcodon pakaraimensis*** (A. Grupe & T.W. Henkel) Xiao-L. He, Di Wang & W.H. Peng, **comb. nov.**

MycoBank: MB 849990

*Basionym*: *Sarcodon pakaraimensis* A.C. Grupe & T.W. Henkel, *Mycologia* **107**: 593 (2015).

***Neosarcodon portoricensis*** (A.C. Grupe & T.J. Baroni) Xiao-L. He, Di Wang & W.H. Peng, **comb. nov.**

MycoBank: MB 849991

*Basionym*: *Sarcodon portoricensis* A.C. Grupe & T.J. Baroni, *Mycologia* **107**: 596 (2015).

***Neosarcodon quercophilus*** (A.C. Grupe & Lodge) Xiao-L. He, Di Wang & W.H. Peng, **comb. nov.**

MycoBank: MB 849992

**Basionym.** *Sarcodon quercophilus* A.C. Grupe & Lodge, *Mycologia* **107**: 600 (2015).

***Neosarcodon umbilicatus*** (A.C. Grupe, et al*.*) Xiao-L. He, Di Wang & W.H. Peng, **comb. nov.**

MycoBank: MB 849993

*Basionym*:*. Sarcodon umbilicatus* A.C. Grupe*et al.*, *Mycologia* **107**: 602 (2015).

***Neosarcodon atroviridis*** (Morgan) Xiao-L. He, Di Wang & W.H. Peng, **comb. nov.**

MycoBank: MB 849994

*Basionym*: *Hydnum atroviride* Morgan, *J. Cincinnati Soc. Nat. Hist.* **18**: 38 (1895).

***Neosarcodon rufobrunneus*** (A.C. Grupe & Vasco-Pal.) Xiao-L. He, Di Wang & W.H. Peng, **comb. nov.**

MycoBank: MB 849995

*Basionym*: *Sarcodon rufobrunneus* A.C. Grupe & Vasco-Pal, *Mycologia* **108**: 792 (2016).

***Neosarcodon pallidogriseus*** (A.C. Grupe & Vasco-Pal.) Xiao-L. He, Di Wang & W.H. Peng, **comb. nov.**

MycoBank: MB 849996

*Basionym*: *Sarcodon pallidogriseus* A.C. Grupe & Vasco-Pal.,, *Mycologia* **108**: 797 (2016).

***Neosarcodon bairdii*** (A.C. Grupe & Vasco-Pal.) Xiao-L. He, Di Wang & W.H. Peng, **comb. nov.**

MycoBank: MB 849997

*Basionym*: *Sarcodon bairdii* A.C. Grupe & Vasco-Pal., *Mycologia* **108**: 799 (2016).

***Neosarcodon colombiensis*** (A.C. Grupe & Vasco-Pal.) Xiao-L. He, Di Wang & W.H. Peng, **comb. nov.**

MycoBank: MB 849998

*Basionym Sarcodon colombiensis* A.C. Grupe & Vasco-Pal., *Mycologia* **108**: 801 (2015).

## DISCUSSION

*Sarcodon* and *Hydnellum* species are common in markets in Sichuan and Yunnan Provinces in Southwest China, and they are of important economic value to the local people (Fig. 1). In addition, some species of the two genera, including *S. imbricatus* and *S. leucopus*, are reputed to have important medicinal functions (Ma et al. 2014; Tan et al. 2020). In the present study, the species diversity of the two genera marketed in Southwest China was analyzed based on morphological characters and DNA sequences (ITS, RPB2 and nLSU). Five species separated from the *S. imbricatus* complex and three of *Hydnellum* are described in this work as new; three new Chinese records of *Hydnellum* and the notable *S. leucopus* are also presented.

The molecular analyses strongly supported the “*Neosarcodon*” clade defined by Larsson et al. (2019), as well as the monophyly of the much reduced “*Sarcodon*” clade and the revised *Hydnellum*. According to the descriptions and illustrations of members in the *Neosarcodon* clade (Grupe et al. 2015, 2016), *Neosarcodon* differs from *Sarcodon* in the adnate hymenophore, its indistinct odor, and the absence of hymenial cystidia. Based on the morphological and molecular evidence, *Neosarcodon* is formally introduced as a distinct genus here. Phylogenetic analyses also showed that the species diversity of *Sarcodon* and *Hydnellum* in the markets was much higher than previously thought, and at least 17 phylogenetic taxa of the two genera could be found in the free markets in Southwest China. Five (*H. illudens*, *H. martioflavum*, *H. versipelle*, *S. imbricatus* and *S. leucopus*) of the 17 species are shared with other continents.

Although *Sarcodon* is one of the most important wild edible mushrooms in Southwest China, it is rather difficult to find these mushrooms in the field for research due to their occurrence in subalpine forests and comprehensive collection by local people. Prior to this study, fewer than 50 taxa were included in the reduced concept of *Sarcodon*, and only two names (*S. imbricatus* and *S. leucopus*) were widely used in China. In the present molecular analyses, nine *Sarcodon* taxa were recovered in China, all of which could be found in the markets sold as edible (marked with black stars ★ in the tree). Surprisingly, seven taxa were included in the traditional *S. imbricatus* complex in addition to *S. imbricatus*: *S. flavidus*, *S. giganteus*, *S. nigrosquamosus*, *S. peseudoimbricatus*, and *S. subsquamosus* which are formally described here; as well as two unnamed *Sarcodon* species that have been temporarily shelved due to the lack of sufficient knowledge about these species. Among these edible *Sarcodon* species, *S. leucopus* can be easily separated from the others by its almost smooth pileus and rather bitter taste, and this species is rather popular in Yajiang County, Sichuan province*.* Members in the *S. imbricatus* complex are mixed together in the markets, where they are usually called “hei hu zhang” or “zhang zi jun”*.* According to the present investigation and molecular analyses on 132 specimens collected in markets, the true *S. imbricatus* is actually the most commonly sold species in the markets, while *S. giganteus* is restricted to Liangshan Yi Autonomous Prefecture in Sichuan Province. Although these species are similar to *S. imbricatus* in macro-morphology, the mature basidiomes exhibit several subtle but stable differences. *S. nigrosquamosus* has dense, small, and almost black scales; *S. flavidus* has a yellow pileal surface; the rather large basidiomata, grayish white pileus, and scales are distinguishing characters for *S. giganteus; S. neosquamosus* possesses a reddish brown pileus; and *S. pseusoimbricatus* is unique in its pale and sparse scales. However, the young basidiomes of *S. giganteus*, *S. neosquamosus*, *S. nigrosquamosus*, and *S. imbricatus* are all rather similar to each other and cannot be distinguished based on morphological characters alone. In the literature, *S. squamosus* and *S. aspratus* are always linked with *S. imbricatus*; in addition, they are often synonymized with *S. imbricatus*, and little information about them has been documented. From the limited information in the literature, it is known that *S. squamosus* grows in pine forests, and *S. aspratus* grows in *Quercus* forests (Johannesson et al. 1999; Vizzini et al. 2013). In the study of Vizzini et al. (2013), *S. squamosus* and *S. aspratus* were shown to be independent species distinct from *S. imbricatus*, and another species (*S. quercinofibulatus*) occurred under *Quercus* that was also separated from the *S. imbricatus* complex based on ITS sequences. Sequence analyses showed that *S. quercinofibulatus* and *S. squamosus* were different from the Chinese collections. In GenBank, only two ITS sequences (DQ448877 and AF335110) were labeled as *S. aspratus*, but these sequences were of low quality and were not included in the present analyses. Judging from the two sequences, they were also different from the Chinese collections.

*Hydnellum* is an important genus of stipitate hydnaceous fungi. *H. concrescens* and *H. cumulatum* were reported as edible in China (Wu et al. 2019), but the present analyses did not reveal their occurrence in China. A recent study (Mu et al. 2021) presents 11 new species of *Hydnellum* with woody basidiomata from China that are rarely found in markets. *Hydnellum* collections sold in the markets are usually more fleshy than other *Hydnellum* species, and many of them were thought to be *Sarcodon* s. lat. in the traditional sense. In the present investigation, eight *Hydnellum* species were found in the markets in Southwest China (marked with black triangles ▲ in the tree). Five of these edible *Hydnellum* species (*H. edulium*, *H. illudens*, *H. lidongensis*, *H. subscabrosellum* and *H. grosselepidotum*), are placed in the clade consisted of *H. scabrosum* and relatives (*Hydnellum* subgenus *Scabrosum* in Mu et al. 2021). All the known species in this clade were placed in *Sarcodon* prior to Larsson et al. (2019). The macromorphological characters of this group are more distant from the typical *Hydnellum* than *Sarcodon* s. str., and they are difficult to separate from *Sarcodon* sometimes. However, morphologically, except for the lack of a distinct smell, bluish or greenish colors in the stipe base and smaller basidiospores can separate members in this group from *Sarcodon* s.str. Phylogenetically, the monophyly of this group is also strongly supported. Although it seemes that subg. *Scabrosum* is a distinct group different from *Sarcodon* and typical *Hydnellum*, treating it as a distinct genus would make the remaining *Hydnellum* paraphyletic. The other three edible *Hydnellum* species in China, *H. subalpinum*, *H. martioflavum* and *H. versipelle*, are distant from those in subgenus *Scabrosum*, and they are placed in three different clades in the present analysis. Further studies based on more samples might further change the circumscription of *Hydnellum* and its infrageneric classification in the future.

## CONCLUSION

In this study, species diversity of the traditional *Hydnellum* and *Sarcodon* species marketed in Southwestern China was analyzed based on morphological and molecular evidence (ITS, nLSU and RPB2). Species diversity of *Sarcodon* and *Hydnellum* in the markets is much higher than previously thought, and 17 phylogenetic species are recovered in the present analyses. Eight new species of *Hydnellum* and *Sarcodon*, and three new Chinese records of *Hydnellum* are added to the list of edible stipitate hydnoid fungi in China. Furthermore, *Neosarcodon* is formally established as a genus, and the generic circumscription of *Sarcodon* is revised based on the combined morphological and molecular evidence.

## Data Availability

All sequence data generated for this study can be accessed via GenBank: https://www.ncbi.nlm.nih.gov/genbank/. All alignments for phylogenetic analyses were deposited in TreeBASE (http://purl.org/phylo/treebase/phylows/study/TB2:S30772).
